# The Expression of HIV-1 Vpu in Monocytes Causes Increased Secretion of TGF-β that Activates Profibrogenic Genes in Hepatic Stellate Cells

**DOI:** 10.1371/journal.pone.0088934

**Published:** 2014-02-13

**Authors:** Paresh Patel, Nabab Khan, Manjusha Rani, Deepti Gupta, Shahid Jameel

**Affiliations:** Virology Group, International Centre for Genetic Engineering and Biotechnology, New Delhi, India; Meharry Medical College, United States of America

## Abstract

There is faster progression to fibrosis in persons with liver injury who are also infected with HIV. Other reports have suggested that HIV can directly infect and activate stellate cells, and the viral Tat and gp160 proteins also induce profibrogenic factors from peripheral blood mononuclear cells (PBMCs). We tested the role of HIV-1 Vpu accessory protein in promoting profibrogenic activation of hepatic stellate cells. Human stellate LX2 cells were cocultured with human monocytic U937 cells stably expressing the Vpu protein or latently infected U1 cells knocked down for Vpu expression, LX2 cells were also cultured with the supernatants from these cells. The expression of profibrogenic markers was evaluated in LX2 cells usingquantitative reverse transcription polymerase chain reaction (qRT-PCR),western blotting, immunofluorescence,flow cytometry and ELISA were used to confirm and quantitate protein expression. Monocytic cells expressing Vpu increased the expression of profibrogenic markers in LX2 cells. The culture supernatants of these cells contained increased levels of transforming growth factor beta (TGF-β), which correlated with increased activity of the AP-1 transcription factor. Antibodies against TGF-β or a TGF-β receptor inhibitor (SB431452) reversed Vpu-mediated profibrogenic activation of LX2 cells, suggesting that TGF-β mediated these effects. The cytokine macrophage migration inhibitory factor (MIF) attenuated Vpu-mediated TGF-β secretion and profibrogenic effects on LX2 cells. Besides its other roles in pathogenesis, Vpu is likely to contribute to hepatic fibrosis through this hitherto unknown mechanism.

## Introduction

Human immunodeficiency virus (HIV) infection persists in about 35 million people worldwide. The transmission of HIV occurs through blood and other body fluids and coinfection with other pathogens, which are also transmitted through the same route, is common. About a third of HIV infected people are coinfected with either hepatitis B virus (HBV) or hepatitis C virus (HCV) [1]. The progression of liver damage during chronic hepatitis is enhanced by coinfection with HIV. Patients coinfected with HCV and HIV show increased hepatic fibrosis and necro-inflammatory activity than persons infected with only HCV [2], [3]. The progression of fibrosis correlates with HIV RNA levels, suggesting that HIV can directly affect liver disease in coinfected patients [4]. The prevalence of fibrosis is also higher in HIV infected persons compared to uninfected ones, with HIV infection reported to be associated with ∼50% increase in liver fibrosis [5].

Hepatic fibrosis represents the wound healing response to liver injury from viral or non-viral etiologies and results from an imbalance between the production and dissolution of the extracellular matrix (ECM) [6]. Hepatic stellate cells (HSCs) are the main contributors to fibrosis. These are vitamin A-rich cells that are normally quiescent and produce type IV collagen, which is characteristic of the normal basement membrane. But following liver injury, the HSCs get activated and transform into a proliferative and contractile cell that starts producing type I collagen (COL-1) in the ECM, which is a characteristic of a cirrhotic liver [7]. The activation of HSCs is also associated with increased expression of other fibrosis markers like matrix metalloproteinase 2 (MMP2), transforming growth factor-beta (TGF-β), procollagen type III (PCT-III), alpha smooth muscle actin-1 (αSMA-1) and vascular enodothelial growth factor (VEGF) [8], [9], [10]. Activated HSCs are susceptible to HIV infection and also respond to a variety of stimuli like TGF-β, which promote hepatic fibrosis [11], [12]. The tissue infiltration of immune cells is also associated with liver injury and cells such as macrophages and CD4+ T cells are infected by HIV, resulting in altered secretion of soluble factors that may also promote hepatic fibrosis [13].

During its replicative cycle, HIV-1 expresses proteins with various cellular functions including the four accessory proteins Vif, Vpr, Nef and Vpu, which are dispensable for viral replication *in vitro* but are required for disease progression in the infected host [14]. While the accessory proteins play important roles in pathogenesis, their role in hepatic fibrosis has not been examined. In this study we have focused on Vpu, which is a transmembrane protein that supports the assembly of new virions by reducing cellular CD4 levels and promotes their egress by reducing surface levels of the restriction factor Bst2 (also called Tetherin or CD317) [15], [16]. We show here that human monocytic cells expressing Vpu or infected with HIV-1 secreted increased levels of TGF-β, which in turn promoted the profibrogenic activity of LX2, a human stellate cell line.

## Materials and Methods

### Cell Lines

The human monocytic cell line, U937 and its derivative U1 cells [17], which are chronically infected with HIV-1 NL4-3, were cultured in RPMI containing 10% fetal bovine serum (FBS). The human hepatic stellate cell line, LX2 [12] was maintained in DMEM containing 2% FBS.

### Plasmids and Antibodies

Retroviral vector pQCXIP-VpuGFP [18] was a kind gift from Dr. Edward Barker (Rush University Medical Center, Chicago, USA). The pQCXIP-GFP control vector was made by PCR amplification and subcloning of AcGFP. The retroviral constructs containing shRNA against Vpu [19], [20] and control shRNA were purchased from Origene Technologies (Rockville, MD, USA). The 3xAP1pGL3 reporter plasmid was obtained from Dr Alexander Dent through Addgene (#40342) [21]. Anti-CD4-PerCP (Becton Dickinson, Gurgaon, India), anti-tetherin-APC (Abcam, Cambridge, UK) and anti-TGFβ (R&D Systems, Minneapolis, MN, USA) were used for flow cytometery; anti-GFP, anti-GAPDH (Santa Cruz Biotechnology, CA, USA) and anti-Vpu antisera [22] (NIH AIDS Reagent Bank, Frederick, MD, USA) were used for western blotting.

### LX2 Coculture

LX2 cells (0.5 million per well) were seeded in 6-well plates. After 24 hr equal numbers of U937 cells expressing either VpuEGFP or EGFP, or U1 cells expressing the Vpu or scrambled shRNA, were added over the LX2 cells in serum free 1∶1 DMEM:RPMI medium followed by incubation for 48 hr. For inhibition experiments, 10 µg/ml anti-TGFβ pan-tropic antibodies (R&D Systems) or an isotype control was added. The TGFβ receptor inhibitor, SB431452 (Sigma-Aldrich, St. Louis, MO, USA) was used at 10 µM [23] and MIF (R&D Systems) was used at 100 ng/ml.

### RNA Isolation and Real Time PCR

RNA was isolated using TRIZOL (Life Technologies, Bangalore, India) according to manufacturer’s instructions. The U937 and U1 cells were seeded in serum-free RPMI for 16 hr and the LX2 cells were washed with PBS before RNA isolation. After quantitation on a NanoDrop (Thermo Scientific, West Palm Beach, FL, USA), 2 µg RNA was used to prepare cDNA with MuLV-RT (Promega, Madison, WI, USA). The real-time assay was performed on a Step One Plus machine (Life Technologies) using the EvaGreen dye (Solis-BioDyne, Tartu, Estonia). All normalizations were done using β -actin levels and the fold changes were calculated by the ΔΔCT method. For qRT-PCR, the following primers were used - MMP2 (NG_008989): Forward GCCCCAGACAGGTGATCTTG, Reverse GCTTGCGAGGGAAGAAGTTGT; TGF- β (NG_013364): Forward, AAGTGGACATCAACGGGTTC, Reverse, 5′ GGTCCTTGCGGAAGT CAATG; COL-1 (NG_007400): Forward, TGCCGTGACCTCAACATGTG, Reverse, GCCGA ACAGACATGCCTG; VEGF (NM_005429): Forward, CAACATCACCATGCAGATTATGC, Reverse, GCTTTCGTTTTTGCCCCTTT; PCT III (NG_007404): Forward, GTTGACC CTAACCAAGGATG, Reverse, GAAGTTCAGGATTGCCGTAG; α-SMA-1 (NG_011541): Forward, CATCCTCCCTTGAGAAGAGTTA, Reverse, TACATAGTGGTGCCCCCTGATA; and β-actin (NG_007992): Forward, ACCAACTGGGACGACATCGAGAAA, Reverse, TAGCACAGCCTGGATAGCAACGTA.

### Retroviral Transduction

The retroviral constructs were cotransfected along with pMLV-GagPol and pVSVG in 293T cells and 48 hr post transfection the retroviral supernatants were collected and the U937 or the U1 cells were transduced for 4 hours. After 48 hr the cells were selected in 350 ng/ml Puromycin for 20 days.

### Flow Cytometry

For surface staining, 1×10^6^ U937 stable cells were washed twice with PBS and fixed with 4% formaldehyde, followed by staining with either 1 µg/ml anti-CD4-PerCP (Becton Dickinson, Gurgaon, India) or 1 µg/ml anti-tetherin-APC (Abcam, Cambridge, UK). For total staining, the U937 or the LX2 cells were fixed as earlier and permeabilized with 0.4% Triton-X100 followed by staining with either 1 µg/ml anti-TGFβ (R&D Systems) ) or 1∶1000 diluted anti-collagen type I, clone 5D8-G9 (EMD Millipore), respectively and 1∶1500 diluted anti-rabbit Alexa 647 (Life Technologies, Bangalore, India). The cells were acquired using a Dako Cyan flow cytometer (Beckman Coulter, Brea, CA, USA) and the data analyzed with FlowJo (Treestar Inc, Ashland, OR, USA).

### Western Blotting

The cells were harvested and lysed in lysis buffer (20 mM Tris pH 7.5, 150 mM NaCl, 1 mM EDTA, 1 mM EGTA, 1% Triton X-100, 2.5 mM sodium pyrophosphate, 1 mM Na_3_VO_4_ and protease inhibitor cocktail [Roche]). The proteins were separated by 12% SDS-PAGE, transferred to either 0.44-micron nitrocellulose membrane (for GFP or GAPDH) or 0.22-micron PVDF membrane (for Vpu), and western blotted with the relevant antibodies. The signals were acquired on a FlourChem M (ProteinSimple, Santa Clara, CA, USA).

### Confocal Microscopy

The LX2 cells were seeded at appropriate density on cover slips in a 12-well plate. The coculture with U937-VpuGFP and U937-GFP cells was performed as described above; 48 hr later the U937 cells were removed, the LX2 cells on coverslips washed with PBS, fixed with 4% paraformaldehyde and permeabilized with 0.4% Triton-X100. The cells were then stained with anti-SMA1, cloneASM-1 (EMD Millipore) and 1∶1500 diluted anti-rabbit Alexa 568 (Life Technologies, Bangalore, India), and mounted using antifade containing DAPI (Invitrogen, Carlsbad, CA, USA). Images were acquired using a Nikon A1/R confocal microscope at 60X magnification. The post acquisition processing and analysis of the images was performed using the NIS Elements software (Nikon, Japan).

### Promoter Luciferase Assay

In a 12-well plate, 0.5×10^6^ HEK293T cells were transfected with 500 ng each of plasmids 3xAP1pGL3 and the Vpu expression plasmid pSGI-Vpu-HA using the jetPRIME transfection reagent (Polyplus Transfection, Illkirch, France). After 48 hr, the cells were harvested and the luciferase activity was measured in lysates using the Dual-Luciferase® Reporter Assay System (Promega, Madison, WI, USA) as per manufacturer’s protocol. Plasmid pRLTK (100 ng) expressing Renilla luciferase was cotransfected and used for normalization.

### TGF-β ELISA

One million cells were seeded in triplicate in the wells of a 12-well plate in 1 ml serum-free RPMI for 24 hr. From this, 100 µl culture supernatants were used for ELISA (eBioscience Inc, San Diego, CA, USA) as per manufacturer’s guidelines.

### Statistical Analysis

Statistical significance was calculated from at least three biological replicates in each experiment using students T-test and p<0.05 was considered significant.

## Results

### Generation of Monocyte Cell Lines Expressing HIV-1 Vpu

**Figure 1:**
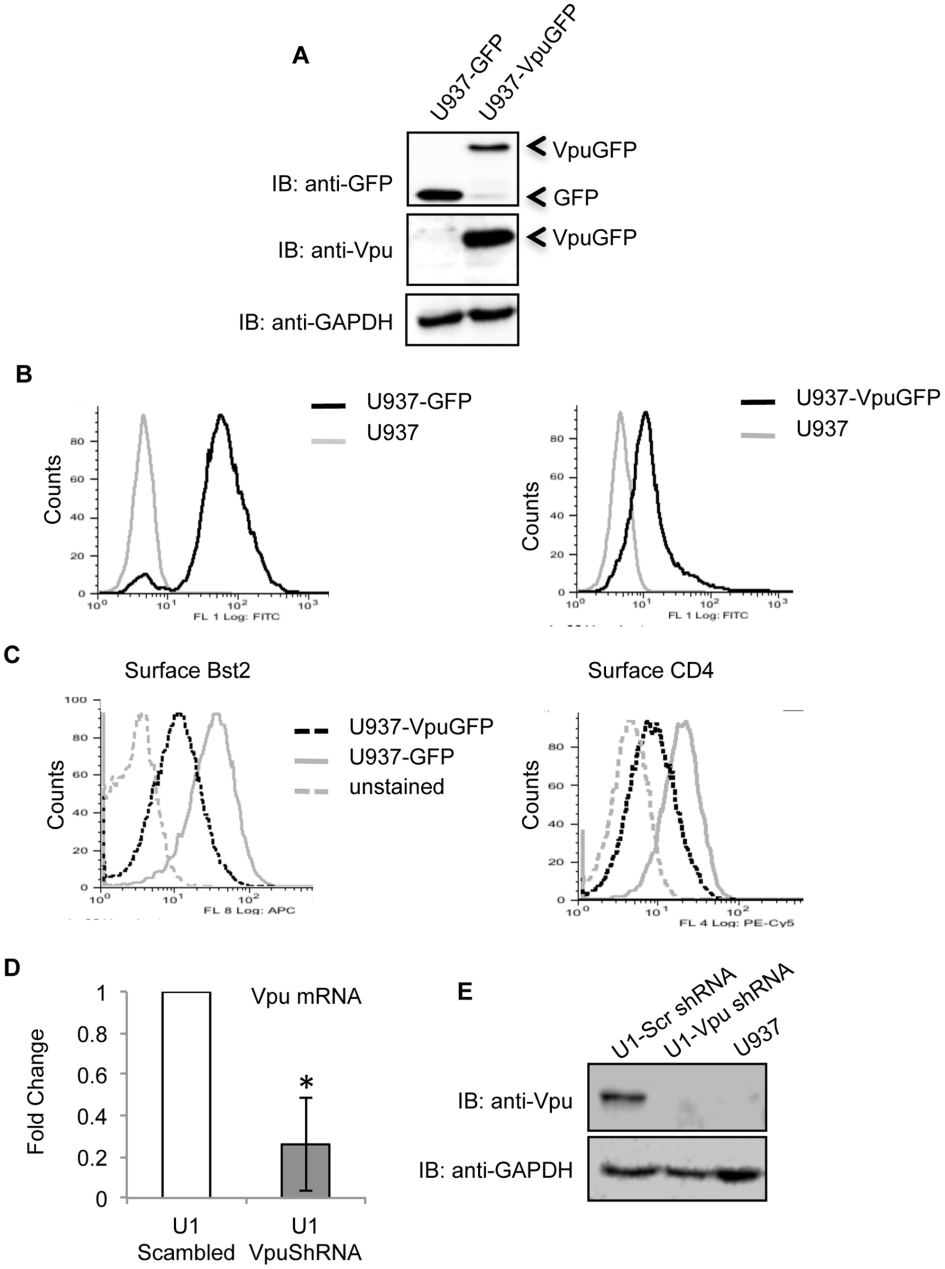
Characterization of Vpu-expressing cell lines. (A) Western blots of lysates of U937-VpuGFP and U937-GFP stable cell lines using anti-Vpu and anti-GFP antibodies. (B) Flow cytometric analysis of GFP expression in U937-VpuGFP and U937-GFP stable cell lines. (C) Flow cytometric analysis of cell surface BST2 and CD4 expression on U937-VpuGFP and U937-GFP stable cell lines. (D) Quantitative RT-PCR of Vpu mRNA levels in U1 cells stably expressing either a Vpu-specific shRNA or a control scrambled shRNA. Error bars represent mean ± SD, n = 3 (*p<0.05). E) Western blot of cell lysates from U1 cells stably expressing either a Vpu-specific shRNA or a control scrambled shRNA, with anti-Vpu antibodies. GAPDH served as a loading control.

We established human monocyte U937 cell lines stably expressing either a VpuGFP fusion protein or GFP. This was confirmed by western blotting (Fig. 1A) and flow cytometry (Fig. 1B). The transmembrane and cytoplasmic domains of Vpu are required for the degradation of Bst2 and CD4, respectively. To ensure that the VpuGFP protein was functional, the levels of Bst2 and CD4 were estimated in the stable cell lines. As expected, there was reduced surface expression of both Bst2 and CD4 in U937-VpuGFP cells compared to U937-GFP cells (Fig. 1C). We also knocked down Vpu expression in U1 cells, which are U937 human monocytic cells latently infected with HIV-1. Retroviruses expressing either a Vpu-specific or scrambled shRNA were used to transduce U1 cells and stable clones were selected. Cells transduced with the Vpu shRNA showed significantly lower levels of Vpu mRNA (Fig. 1D) and protein (Fig. 1E). Thus, we have established stable human monocytic cell lines that either express Vpu or are Vpu-deficient in the provirus background.

### Vpu-expressing Monocytes Activate Profibrogenic Markers in Stellate Cells

**Figure 2:**
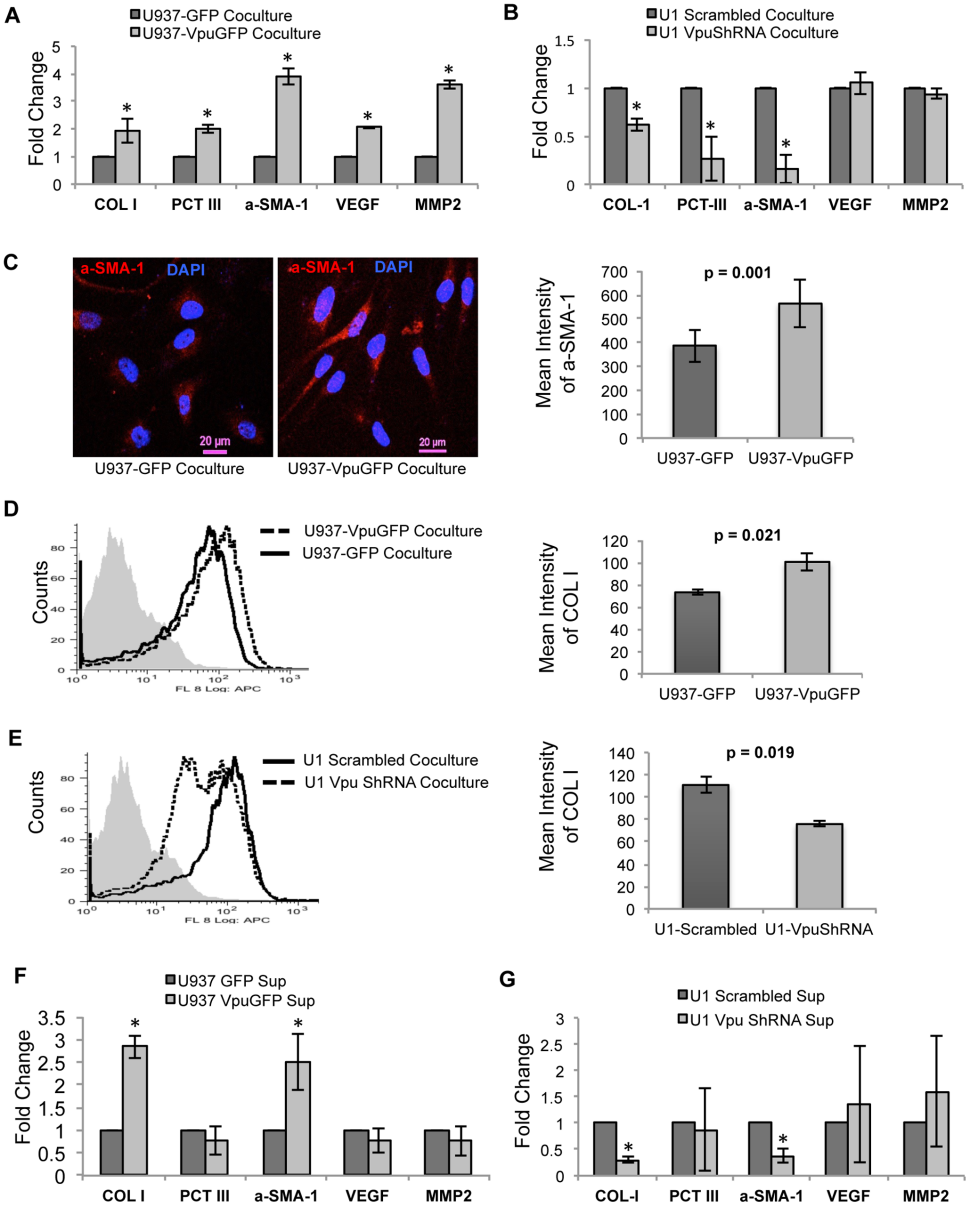
Profibrogenic effects of HIV-1 Vpu expressing monocytes on human hepatic stellate LX2 cells. The LX2 cells were cocultured with (A) U937-VpuGFP and U937-GFP cells or (B) U1-Scrambled and U1-VpuShRNA cells, as described in Methods. The expression levels of the indicated mRNAs in the LX2 cells were then estimated by qRT-PCR. All expression values were normalized to those in the control cells, n = 3. (C) Confocal microscopic analysis of intracellular αSMA-1 expression in LX2 cells cocultured with either U937-GFP or U937-VpuGFP cells. The bar plot on the right represents the mean intensity of αSMA-1 in the LX2 cells, n = 32. Flow cytometric analysis of intracellular COL-1 levels in LX2 cells cocultured with (D) U937-VpuGFP and U937-GFP cells or (E) U1-Scrambled and U1-VpuShRNA cells. The bar plot on the right represents the mean fluorescence intensity (MFI) of COL-1 in the LX2 cells, n = 3. The LX2 cells were treated with culture supernatants from (F) U937-VpuGFP and U937-GFP cells or (G) U1-Scrambled and U1-VpuShRNA cells, as described in Methods. The expression levels of the indicated mRNAs in the LX2 cells were then estimated by qRT-PCR. All expression values were normalized to those in the control cells, n = 3. Error bars represent mean ± SD (*, p<0.05).

We hypothesized that Vpu-expressing monocytes may increase the profibrogenic activity of human stellate cells. To address this, U937-VpuGFP or U937-GFP cells were co-cultured with LX2 cells for 48 hr, following which the former were removed and the latter were assayed for the expression of profibrogenic markers by qRT-PCR. Compared to control, LX2 cells co-cultured with U937-VpuGFP showed increased expression of COL-1, PCT-III, αSMA-1, VEGF, and MMP2 mRNAs (Fig. 2A). Similarly, LX2 cells co-cultured with U1 cells that did not express Vpu showed reduced expression of COL-1, PCT-III and αSMA-1, but the expression of VEGF and MMP2 mRNAs remained unaltered, possibly due to the effects of other HIV-1 proteins on their regulation (Fig. 2B). On confocal microscopy and flow cytometric analyses, LX2 cells cocultured with U937-VpuGFP showed higher intracellular levels of αSMA-1 and COL-1 proteins as compared to those cocultured with U937-GFP cells (Fig. 2C and 2D). Similarly, flow cytometric analysis of LX2 cells cocutured with U1 cells knocked down for Vpu showed reduced intracellular COL-1 levels (Fig. 2E). These effects of Vpu-expressing monocytes on stellate cells can be attributed to either cell-to-cell contact or to factors secreted by the Vpu-expressing cells. To test this, clarified culture supernatants were used to stimulate LX2 cells. Compared to control, culture supernatants from U937-VpuGFP cells led to increased expression of COL-1 and αSMA-1 in the LX2 cells (Fig. 2F). Similarly, LX2 cells treated with supernatants from U1 cells knocked down for Vpu expression showed reduced expression of COL-1 and αSMA-1 (Fig. 2G). While there was variability in activated markers between the co-culture and culture supernatant models, increased fibrogenic activation of LX2 cells was observed when Vpu was expressed within the stimulating monocytes.

### Vpu Expressing Monocytes Activate Stellate Cells through Increased TGF-β Production

**Figure 3:**
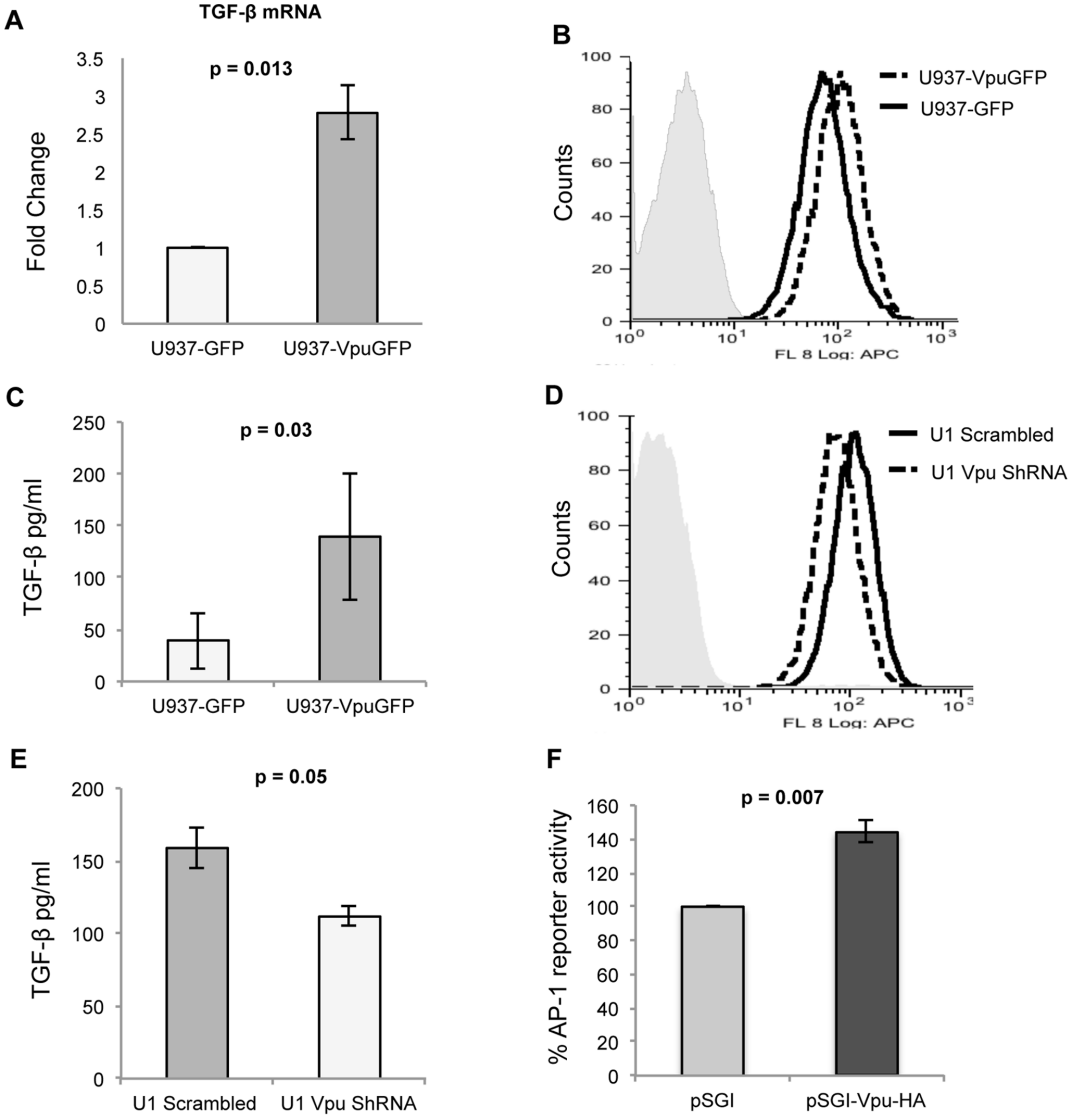
Vpu expressing monocytes show increased levels of TGF-β. (A) Quantitative RT-PCR of TGF-β mRNA levels in U937-GFP and U937-VpuGFP cells, expressed as fold-change in the latter compared to the former. (B) Flow cytometric analysis of TGF-β expression in U937-VpuGFP and U937-GFP stable cell lines. (C) Quantitation of TGF-β secreted in the culture media of U937-VpuGFP and U937-GFP stable cell lines. (D) Flow cytometric analysis of TGF-β expression in U1-scrambled and U1-VpuShRNA stable cell lines. (E) Quantitation of TGF-β secreted in the culture media of U1-scrambled and U1-VpuShRNA stable cell lines. (F) Luciferase reporter quantitation of AP-1 activity in HEK293T cells transfected with expression vector pSGI-Vpu-HA compared to the empty pSGI-HA vector. Error bars represent mean ± SD from three independent experiments.

Since earlier studies have shown TGF-β to promote hepatic fibrosis [24], we tested whether this mediated the effects of Vpu-expressing cells. The U937-VpuGFP cells expressed increased intracellular levels of TGF-β mRNA (Fig. 3A) and protein (Fig. 3B) and secreted higher levels of TGF-β in the culture supernatant (Fig. 3C). Similarly, compared to U1-scrambled cells, the U1-VpuShRNA cells showed reduced intracellular (Fig. 3D) and secreted (Fig. 3E) levels of TGF-β. These data confirm that Vpu expression in U937 cells led to increased expression and secretion of TGF-β. Previous reports also suggest that TGF-β synthesis is transcriptionally regulated by AP-1 binding to its cognate motif within the TGF-β gene promoter [25], [26]. To test if Vpu increased AP-1 activity, we used a reporter construct in which luciferase expression is driven by a minimal promoter and 3X AP-1 binding motifs. When cotransfected in HEK293T cells, there was increased luciferase expression in the presence of Vpu (Fig. 3F), confirming increased AP-1 activity in Vpu-expressing cells. Thus, the Vpu-mediated increase in TGF-β expression is likely to be due to increased AP-1 activity in these cells.

**Figure 4:**
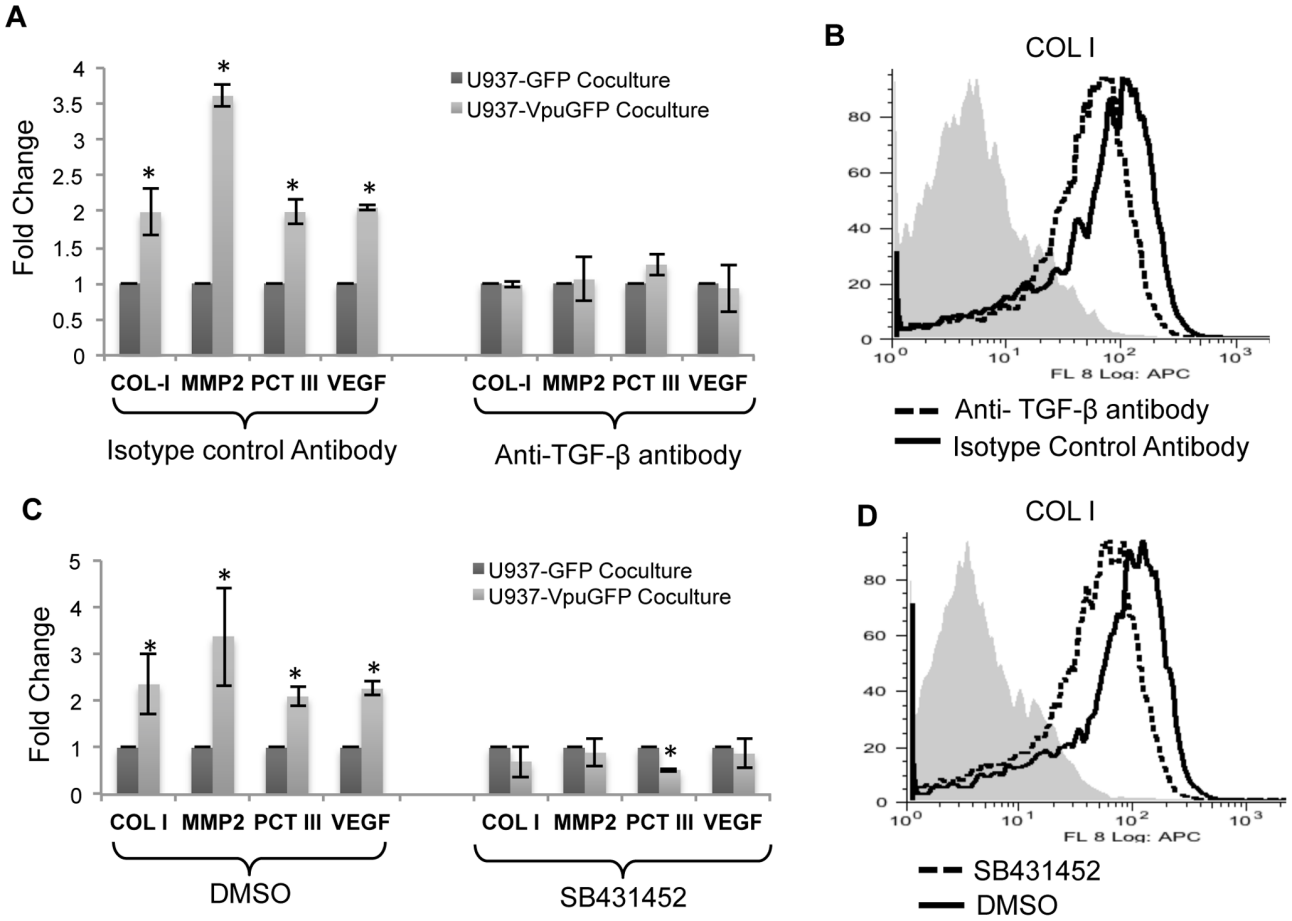
TGF-β secreted from Vpu-expressing monocytic cells is responsible for the profibrogenic effects on LX2 stellate cells. (A) LX2 cells were cocultured with either U937-GFP or U937-VpuGFP cells together with either a pantropic anti-TGFβ antibody or an isotype control antibody as described in Methods. Quantitative RT-PCR was then carried out to estimate the expression levels of mRNAs for COL-1, αSMA-1, PCT-III, MMP2 and VEGF. All expression levels are shown as fold changes normalized to the U937-GFP control. (B) LX2 cells were cocultured with U1-scrambled cells together with either a pantropic anti-TGFβ antibody or an isotype control antibody, and flow cytometry was used to estimate the expression levels of intracellular COL-1. (C) LX2 cells were cocultured with either U937-GFP or U937-VpuGFP cells together with the TGF-β receptor inhibitor SB431452 (or DMSO as control) as described in Methods. Quantitative RT-PCR was then carried out to estimate the expression levels of mRNAs for COL-1, MMP2, PCT-III, and VEGF. All expression levels are shown as fold changes normalized to the U937-GFP control. (D) LX2 cells were cocultured with U1-scrambled cells in the presence of either SB431452 or DMSO, and flow cytometry was used to estimate the expression levels of intracellular COL-1. Error bars represent mean ± SD from three independent experiments; *p<0.05.

We next asked whether TGF-β was responsible for the profibrogenic effects of Vpu-expressing cells on stellate cells. For this, LX2 cells were cocultured with either U937-GFP or U937-VpuGFP cells in the presence of a pantropic TGF-β antibody or an isotype control antibody. As earlier, LX2 cells cocultured with U937-VpuGFP cells showed increased profibrogenic activity, and anti-TGF-β abrogated these effects (Fig. 4A). This was also the case for intracellular COL-1 protein expression in LX2 cells cocultured with U1-scrambled cells (Fig. 4B).

The effects of extracellular TGF-β are manifested on cells when it binds to the cell surface TGF-β receptor and signals downstream of it. We tested the role of TGF-β signaling by coculturing LX2 cells with either U937-GFP or U937-VpuGFP cells in the absence or presence of SB431542, a potent and specific inhibitor of the TGF-β receptor superfamily. The increase in profibrogenic markers in LX2 cells cocultured with U937-VpuGFP cells was abrogated on treatment with SB431542 (Fig. 4C). This was also the case for intracellular COL-1 protein expression in LX2 cells cocultured with U1- scrambled cells (Fig. 4D).

### MIF Inhibits Vpu-mediated Profibrogenic Effects in Stellate Cells

**Figure 5:**
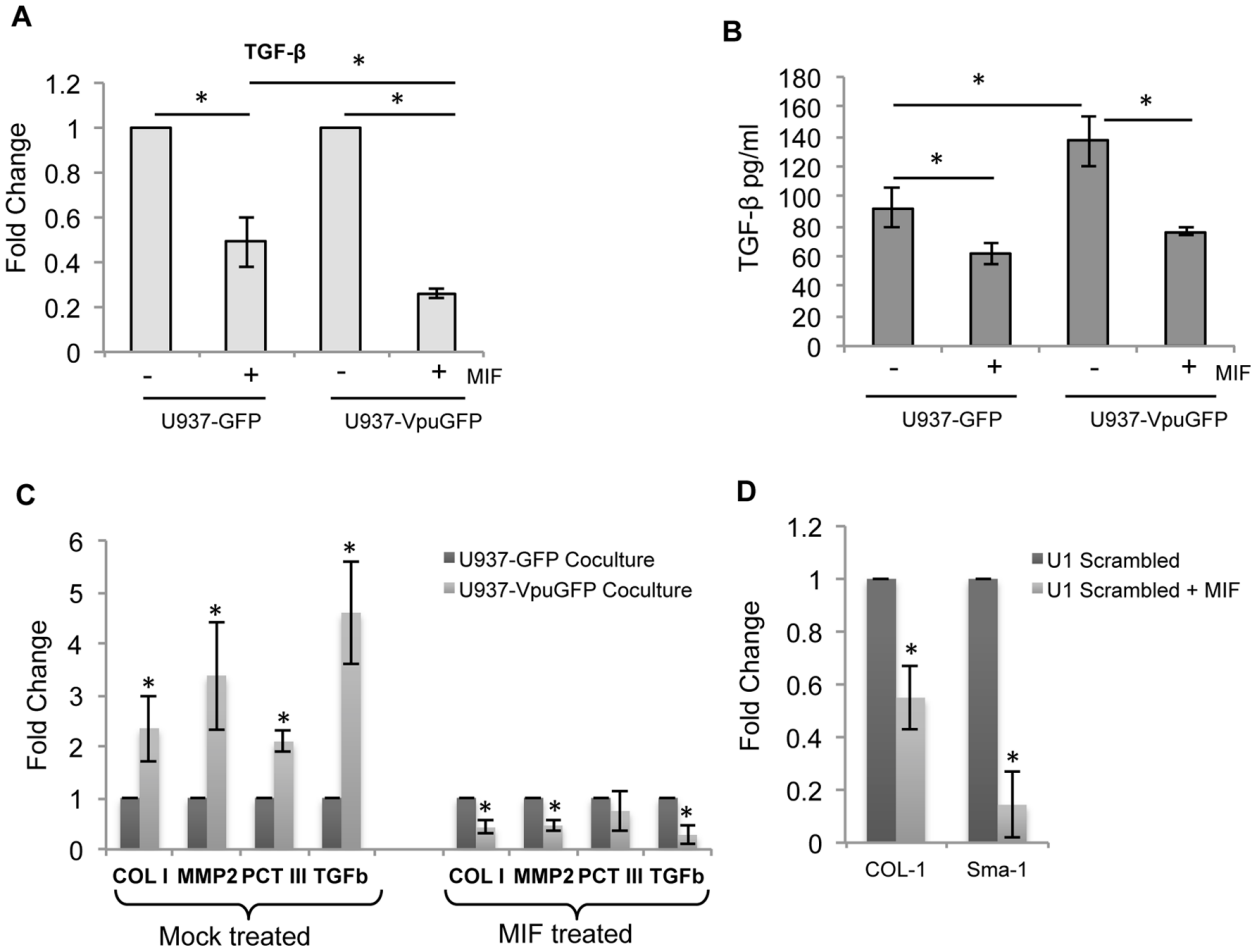
MIF treatment reduces TGF-β in monocytes and ameliorates the profibrogenic effects of Vpu expressing monocytes. (A) Quantitative RT-PCR estimation of TGF-β mRNA levels in U937-GFP and U937-VpuGFP cells treated with MIF. The expression levels are shown as fold changes normalized to the untreated controls. (B) Quantitation of TGF-β in the culture supernatants of the U937-VpuGFP and U937-GFP cultures treated with MIF or untreated controls. (C) LX2 cells were cocultured with either U937-GFP or U937-VpuGFP cells together with MIF as described in Methods. Quantitative RT-PCR was then carried out to estimate the expression levels of mRNAs for COL-1, MMP2, PCT-III and TGF-β in LX2 cells. All expression levels are shown as fold changes normalized to the U937-GFP control. (D) LX2 cells were cocultured with U1-scrambled cells together with MIF, and quantitative RT-PCR was used to estimate the expression levels of COL-1 and αSMA-1 mRNAs; the values are expressed as fold changes normalized to the untreated culture condition. The data are representative of three biological replicates. Error bars represent mean ± SD; *p<0.05.

We have shown earlier that Vpu interacts with CD74 resulting in reduced surface expression of MHCII and attenuation of the immune response to HIV-1 infection [27]. Besides its role as the invariant chain of MHCII, CD74 is also expressed on the cell surface and functions as a receptor for the cytokine macrophage migration inhibitory factor (MIF) [28]. In murine models of fibrosis, MIF was anti-fibrotic; animals deficient for MIF or CD74 had higher risk for hepatic fibrosis [29]. We therefore explored if MIF can mitigate the effects of Vpu-expressing cells on stellate cells. The LX2 cells express detectable levels of surface CD74 (Fig. S1A), and while treatment of these cells with MIF led to reduced TGF-β expression, there was no effect of MIF on COL-1 expression in LX2 cells (Fig. S1B). Treatment with MIF resulted in reduced expression of TGF-β in U937-GFP and U937-VpuGFP cells, the effect being more pronounced in the latter (Fig. 5A). This is also reflected in reduced TGF-β secreted from cells treated with MIF (Fig. 5B). Coculture with U937-VpuGFP cells in presence of MIF led to reduced expression of COL-1, MMP2, PCT-III and TGF-β in LX2 cells (Fig. 5C). This was also confirmed in a coculture of U1-scrambled cells and LX2 cells with or without MIF. Treatment with MIF led to significantly reduced expression of COL-1 and αSMA-1 in LX2 cells (Fig. 5D).

## Discussion

There is faster progression of hepatic fibrosis and earlier development of cirrhosis in HCV-infected persons who are also co-infected with HIV, but not in those patients in whom HIV viremia is under control due to HAART [2], [3]. Although HIV does not infect hepatocytes, the main cell type in the liver, this supports a direct effect on hepatic fibrosis. One recent report showed that HIV directly infects and activates stellate cells, the main cell type that contributes to hepatic fibrosis [11]. Liver injury is also associated with the infiltration of immune cells such as monocytes/macrophages and T cells, which are themselves susceptible to HIV infection and secrete factors that contribute to hepatic stellate cell activation and fibrosis. One such factor is TGF-β, which is increased in the plasma of HIV-infected patients, being contributed mainly by monocytes and CD8+ T-cells among infected PBMCs [30], [31], [32]. Purified HIV Tat and gp160 proteins have also been shown to induce TGF-β expression in human PBMCs [33], [34], [35], but none of the HIV-1 accessory proteins have been studied for this function.

Fibrosis is a chronic condition and the immune cells involved in its promotion might also be chronically infected. Although HIV does not infect a majority of monocytes, cells of the monocyte/macrophage lineage are important latent reservoirs of HIV and these cells also infiltrate the liver during hepatic injury [36], [37]. To study this we used an *in vitro* co-culture model in which either U937 cells stably expressing Vpu or latently infected U1 cells engineered to reduce Vpu expression were used together with LX2 human stellate cells, which on activation expresses type 1 collagen and other fibrogenic markers [12]. The U1 cells produce very small amounts of infectious HIV, which was not sufficient to cause measureable infection of LX2 cells. The levels of HIV RNA in LX2 cells cocultured with U1 cells were below the levels of detection (data not shown). The U937 cells stably expressing Vpu (but not control cells) and the U1 cells (but not those knocked down for Vpu expression) both led to measurable activation of profibrogenic markers in LX2 cells in the co-culture system. This was also the case when culture supernatants of these cells were used on LX2 cells, suggesting that a secretory factor was responsible for this stimulation.

The U937-VpuGFP model was used to create an ‘all or none’ phenomenon wherein we asked whether the expression of Vpu in U937 monocytic cells has any effects on the expression of fibrogenic markers in LX2 human stellate cells when the two were cocultured. Since U937-VpuGFP was a system wherein Vpu was overexpressed in the absence of other HIV-1 proteins, to check the observed effects of Vpu on fibrosis at near-physiological conditions, we have used the U1-Vpu ShRNA model to selectively reduce the expression of Vpu when other HIV-1 proteins were naturally expressed. It is to be noted that U1 cells are also derived from U937 cells, so the backgrounds of the two cellular models are similar.

In a chronic model of dimethylmitrosamine (DMN) induced liver fibrosis, targeting TGF-β signaling with GW6604, an ALK5 inhibitor prevented mortality in mice [38]. Similar effects were observed when TGF-β signaling was blocked by expression of a truncated dominant negative type II TGF-β receptor in DMN treated rats [39]. These observations establish TGF- β as a profibrotic cytokine. The stable expression of Vpu in U937 cells as well as its expression from the provirus in U1 cells led to increased expression and secretion of TGF-β. We used anti-TGF-β antibodies and a chemical inhibitor of the TGF-β receptor superfamily to confirm that TGF-β secreted from monocytic cells mediated the profibrotic effects of Vpu. Previously TGF-β was shown to be important for HIV pathogenesis by promoting virus production and impairing the host immune response [30]. It stimulates transcription from the HIV-1 LTR promoter by activating NFκB through a non-classical mechanism distinct from IκB degradation [40]. There appears to be a complex link between NFκB, TGF-β, Bst2 and Vpu [41]. One important function of Vpu is to counteract restriction of virus release by Bst2, which induces NFκB through the TGF-β-activated kinase 1 (TAK1) downstream of TGF-β binding to its cell surface receptor [40]. The expression of Vpu inhibits Bst2-mediated NFκB induction through a β-TrCP-dependent mechanism [41]. We now show that Vpu induces the expression of TGF-β in monocytes, which is mediated by increased AP-1 activity, shown earlier to be important for TGF-β transcription [25], [26]. The secreted TGF-β activates profibrogenic activity in hepatic stellate cells.

A protective role has been ascribed to MIF in murine models of hepatic fibrosis [29]. We therefore tested this, especially since our earlier work showed Vpu to directly bind CD74, which is one component of the MIF receptor. Analogous to murine hepatic stellate cells, LX2 cells also showed a reduction in TGF-β expression following MIF treatment, potentially attenuating the autocrine loop leading to stellate cell activation. In control as well as Vpu-expressing U937 cells, there was a reduction in TGF-β expression on MIF treatment and this was also reflected in the ability of these cells to induce the expression of profibrogenic markers in LX2 cells. Compared to control cells, U937-VpuGFP cells showed a more pronounced reduction in TGF-β expression following MIF treatment. This could be because Vpu binds and retains CD74 at intracellular sites to reduce its cell surface levels [27]. Our experiments are unable to differentiate between the direct effects of MIF on LX2 cells and indirect effects mediated by reduced secretion of TGF-β from MIF-treated U937 cells. But, they do support the protective effects of MIF on hepatic fibrosis observed earlier in mice. They further show that MIF can overcome Vpu or HIV infection mediated profibrotic effects of monocytes on hepatic stellate cells.

We show that interfering with TGF-β signaling reduces the profibrogenic effects of HIV-1 infected monocytes on hepatic stellate cells *in vitro*. Although further *in vivo* validation of these observations is required, our results indicate a possible strategy for the therapeutic use of MIF and TGF-β signaling inhibitors in patients with HIV-induced liver fibrosis.

## Supporting Information

Figure S1 (A) Flow cytometric analysis of the surface CD74 levels in LX2 cells. The LX2 cells were stained with a primary anti-CD74 antibody and secondary anti-mouse-Alexa-488 antibody, and acquired on a Dako Cyan flow cytometer. (B) LX2 cells were treated with 100 ng/ml MIF for 48 hr, following which RNA was isolated and the expression levels of TGF-β and COL-1 were estimated by qRT-PCR. The levels are expressed as fold changes relative to untreated LX2 cells.
(TIF)
