# Supplementary figures and images for: The Expression of HIV-1 Vpu in Monocytes Causes Increased Secretion of TGF-β that Activates Profibrogenic Genes in Hepatic Stellate Cells
Source: PLoS One. 2014 Feb 13;9(2):e88934. doi: 10.1371/journal.pone.0088934 (PMC3923874; doi:10.1371/journal.pone.0088934)

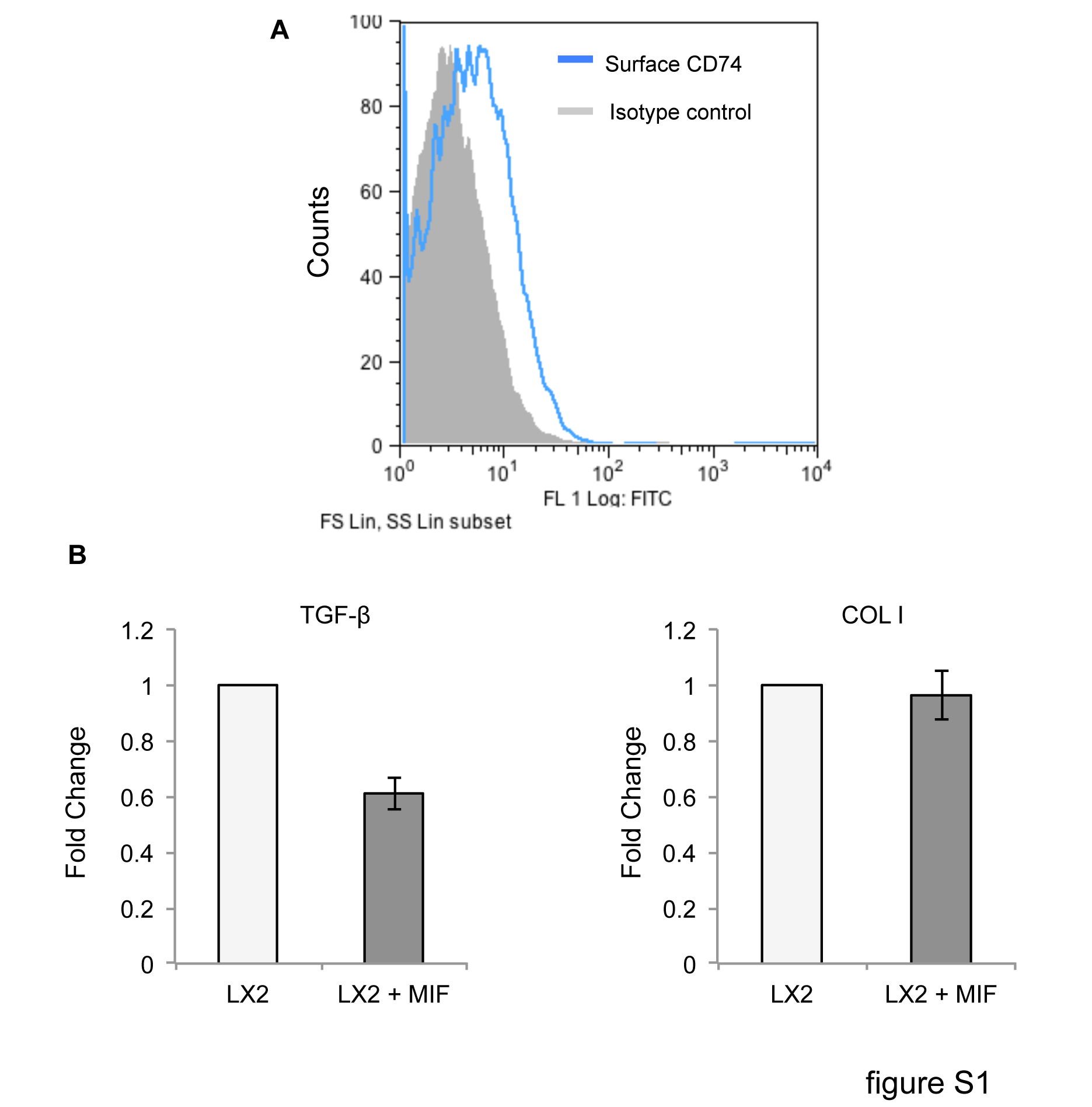

Supplement: Figure S1 — (A) Flow cytometric analysis of the surface CD74 levels in LX2 cells. The LX2 cells were stained with a primary anti-CD74 antibody and secondary anti-mouse-Alexa-488 antibody, and acquired on a Dako Cyan flow cytometer. (B) LX2 cells were treated with 100 ng/ml MIF for 48 hr, following which RNA was isolated and the expression levels of TGF-β and COL-1 were estimated by qRT-PCR. The levels are expressed as fold changes relative to untreated LX2 cells. (TIF) [file pone.0088934.s001.tif]
